# Preparing better: Accelerating COVID-19 Therapeutic Interventions and Vaccines (ACTIV) therapeutics trials lessons learned: A call to the future

**DOI:** 10.1017/cts.2024.178

**Published:** 2024-10-15

**Authors:** Stacey J. Adam, Timothy G. Buchman, Judith S. Currier, Ruxandra Draghia-Akli, Josh P. Fessel, Elizabeth S. Higgs, Eric A. Hughes, Lisa LaVange, Joseph P. Menetski, Sarah W. Read, Yves Rosenberg, Randall Tressler, ACTIV Therapeutics-Clinical Working Group

**Affiliations:** 1 Foundation for the National Institutes of Health, North Bethesda, MD, USA; 2 Critical Care Center, Emory University School of Medicine, Atlanta, GA, USA; 3 Department of Medicine, David Geffen School of Medicine, University of California, Los Angeles, CA, USA; 4 The Janssen Pharmaceutical Companies of Johnson & Johnson, Titusville, NJ, USA; 5 National Center for Advancing Translational Sciences, National Institutes of Health, Bethesda, MD, USA; 6 National Institute of Allergy and Infectious Disease, National Institutes of Health, Bethesda, MD, USA; 7 Teva Pharmaceuticals, Parsippany, NJ, USA; 8 Biostatistics, Gillings School of Global Public Health at the University of North Carolina at Chapel Hill, NC, USA; 9 National Heart, Lung, and Blood Institute, National Institutes of Health, Bethesda, MD, USA

**Keywords:** Covid-19, trials, therapeutics, master protocol, lessons learned

## Abstract

The Accelerating COVID-19 Therapeutic Interventions and Vaccines Therapeutic-Clinical Working Group members gathered critical recommendations in follow-up to lessons learned manuscripts released earlier in the COVID-19 pandemic. Lessons around agent prioritization, preclinical therapeutics testing, master protocol design and implementation, drug manufacturing and supply, data sharing, and public–private partnership value are shared to inform responses to future pandemics.

## Introduction

COVID-19 was declared a pandemic by the World Health Organization (WHO) on March 11, 2020. The rate of global transmission and mortality from its beginning to its declared end on May 18, 2023, raised some of the largest public health, socioeconomic, and scientific challenges in history. COVID-19 manifested widely and severely among older persons, individuals with chronic health problems, and minority populations in the USA, where it ultimately caused at least 1 million deaths despite relatively early access (within the first year of the pandemic) to effective vaccines and therapeutic agents [1]. The initial countermeasure response was swift and sizeable, yet uncoordinated. The Accelerating COVID-19 Therapeutic Interventions and Vaccines (ACTIV) public–private partnership (PPP) was established on April 17, 2020, to coordinate research response efforts [2]. As part of the ACTIV PPP, an investigational therapeutics prioritization effort was established [3], and master protocols were developed [4] to evaluate prioritized therapeutic candidates. These tasks were designed with a portfolio approach to serve specific patient populations.

**Figure 1.**
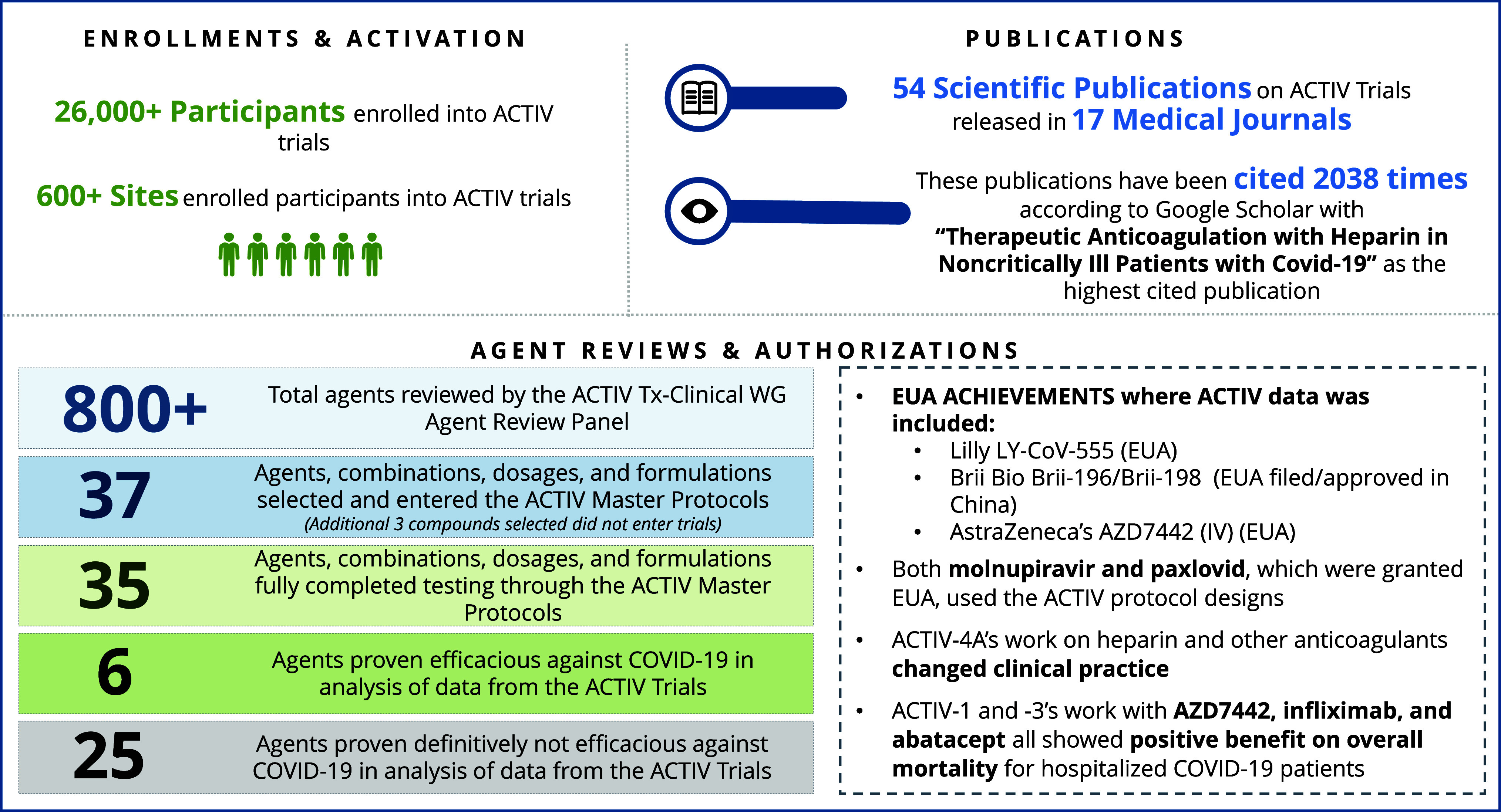
ACTIV therapeutics testing achievements. The ACTIV Therapeutics-Clinical Working Group and master protocols achieved a number of successes that furthered the global understanding of which therapies were and were not beneficial for treating COVID-19. Provided here is a snapshot of the high-level achievements to date of the ACTIV master protocols and their impact on the pandemic, patients, the scientific community, and knowledge of the disease. The figure summarizes the number of participants enrolled, global sites participating in the trials, the number of agents reviewed and tested in the trials, and the current number of journal articles and citations from this work. ACTIV = Accelerating COVID-19 Therapeutic Interventions and Vaccines, ACTIV tx-clinical WG = ACTIV Therapeutic-Clinical Working Group, EUA = emergency use authorization, Lilly = eli Lilly and Company.

**Figure 2.**
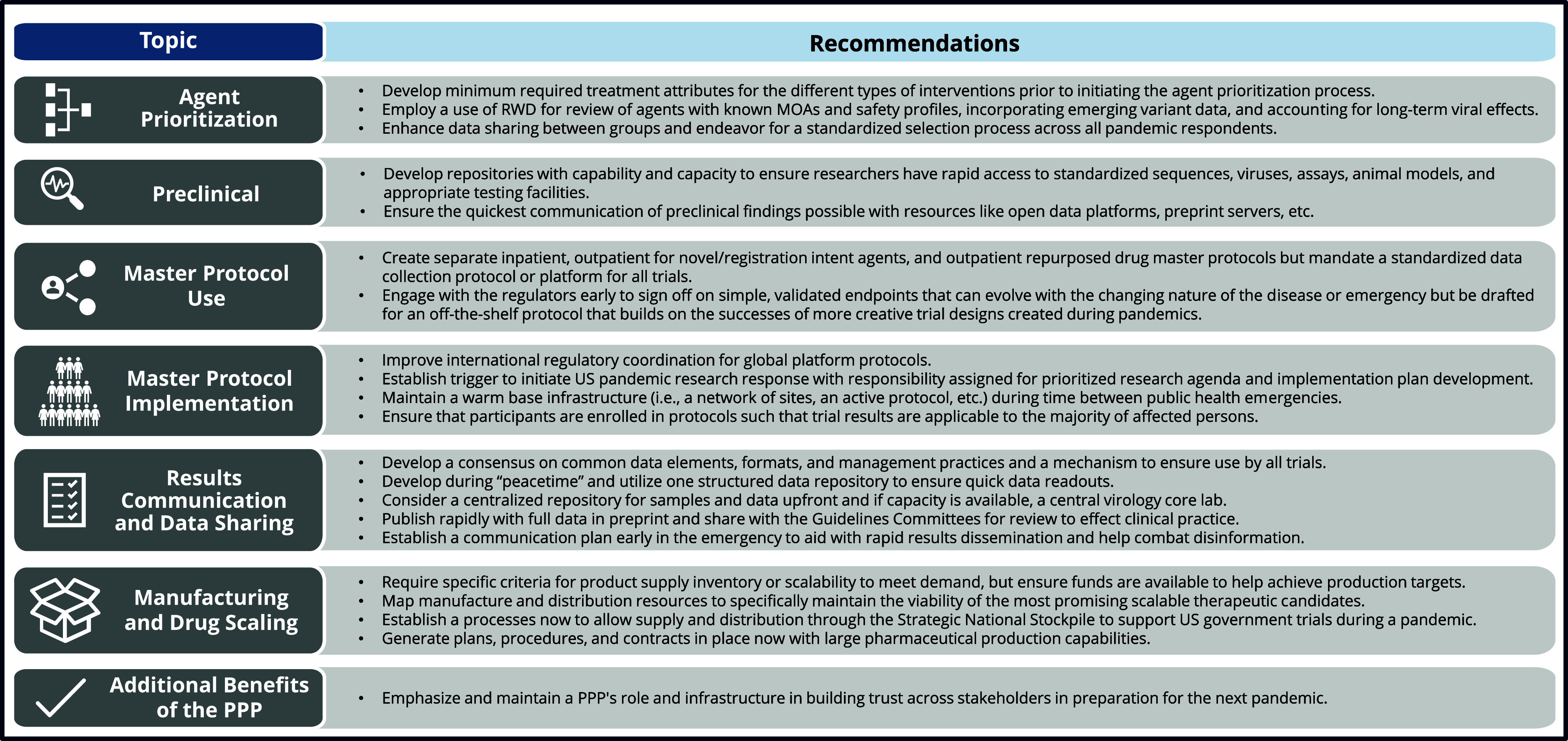
ACTIV therapeutics-clinical working group overarching recommendations for future pandemic responses. The high-level takeaway lessons learned from the ACTIV master protocols can be organized topically in order of the overarching steps of establishing the platform, including prioritizing potential agents, utilizing preclinical data, creating the master protocols, implementing the master protocols, gathering, analyzing, and sharing data, handling logistics of manufacturing and drug scaling, and utilizing the PPP network. These lessons build on those from publications released earlier in the pandemic by the ACTIV Therapeutics-Clinical Working Group. MOAs = mechanisms of action, PPP = publicprivate partnership, RWD = real-word data, US = United States.

To expedite and improve future pandemic responses, the intergovernmental political forum, the Group of Seven (G7), developed an aspiration of the “100-day mission” [5], a gold standard of creating equitable access. This mission addresses many potential improvements recommended by ACTIV and outlines that appropriate “peacetime” preparation is essential to ensure safe and effective medical countermeasures will be available within the first 100 days of a future pandemic. This report and others from the ACTIV teams focus on lessons learned from the effort to accelerate development of therapeutics for COVID-19, which can be applied to future pandemic preparedness and response efforts. This report seeks to convey the advantages and disadvantages from an executive perspective, cataloged by the ACTIV Therapeutics-Clinical and -Preclinical Working Groups (ACTIV TX-Clin and Preclin WGs). The achievements of the ACTIV protocols are summarized in Figure 1 and timelines for important trial milestones summarized in Supplemental Figure 1. Each section addresses and describes key lessons learned for aspects of therapeutic testing within the ACTIV PPP and seeks to detail the rationale for “Recommendations” summarized in Figure 2, meant to aid future groups faced with similar challenges.

## Agent prioritization

**Figure 3.**
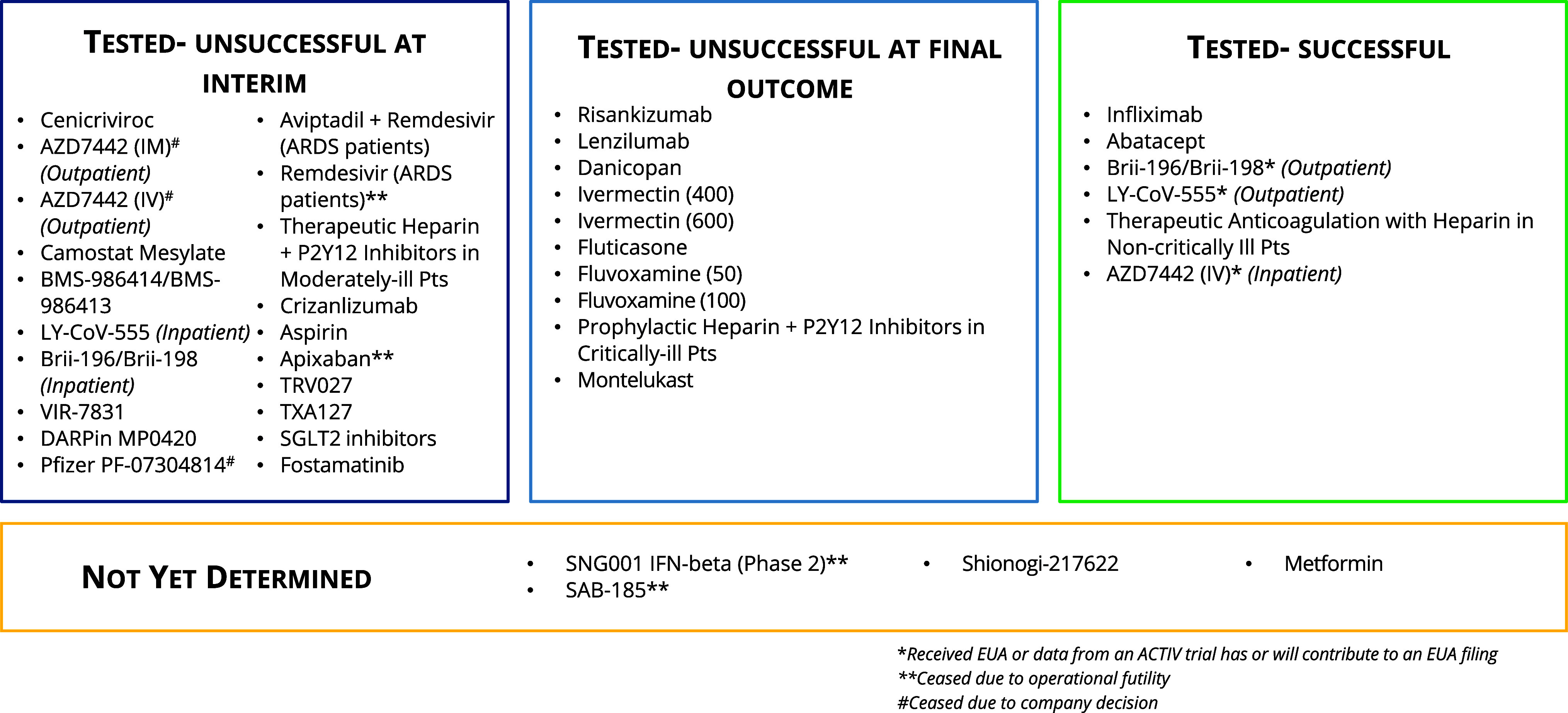
Results of compounds tested in the ACTIV master protocols. Status at the time of report submission of the 37 agents and compounds tested in the ACTIV trials. Determination of an agent as either successful or unsuccessful in one of the master protocols is determined by the completion of the predetermined primary or significant secondary endpoints of trials. The dark blue box on the left reflects the agents tested in any of the 11 ACTIV master protocols that were determined at any interim review to have met the criteria to stop the trial early due to preset futility boundaries; agents that were stopped due to a company decision to no longer pursue the agent; or agents that were ceased due to operational futility causing an in ability to complete enrollment in the given patient population as the pandemic progressed. The light blue box in the middle reflects those agents that completed full enrollment of the prespecified number of patients for their sub-study within the master protocol, but upon final statistical analysis did not achieve significance according to prespecified primary endpoints. The green box in the right reflects the agents that upon testing proved efficacious either by prespecified primary endpoints or significant secondary endpoints within the master protocols. Finally, the yellow box at the bottom reflects those agents that are still undergoing testing within one of the 11 master protocols or statistical analysis by the trial teams and therefore their outcomes are unknown at the time of this report. ACTIV = accelerating COVID-19 Therapeutic Interventions and Vaccines, EUA = emergency use authorization, IM = intramuscular injection, IV = intravenous.

**Figure 4.**
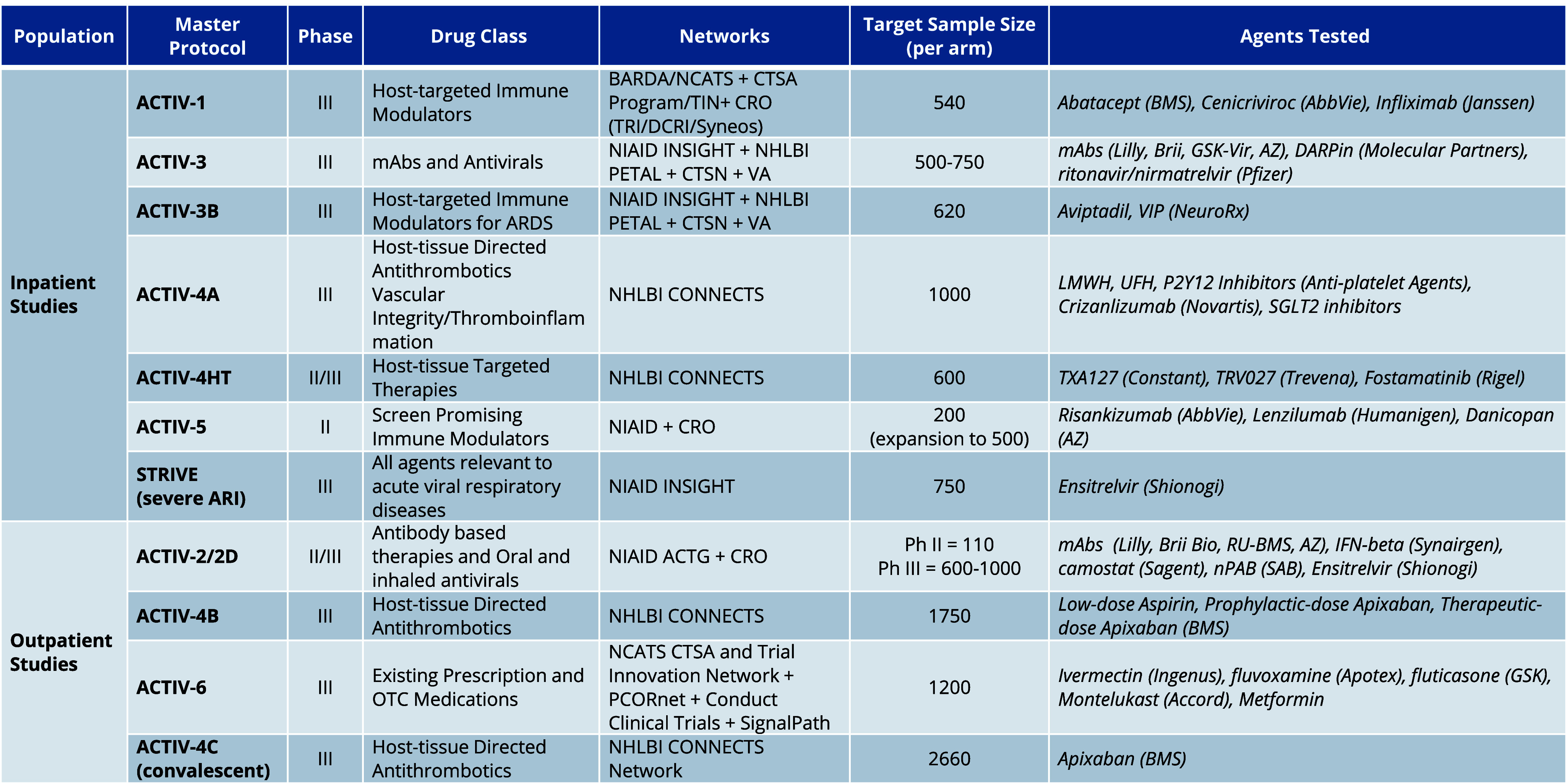
Summary of ACTIV therapeutics master protocols. The 11 ACTIV master protocols tested 37 single agents or combinations. This table summarizes the important design aspects for each protocol, including the patient population studied, phase of the trial, drug class researched, networks leading the conduct of the trial, target sample size for each trial arm, and finally the agents tested. ACTG = AIDS Clinical Trials Group, ACTIV = Accelerating COVID-19 Therapeutic Interventions and Vaccines, ARDS = acute respiratory distress syndrome, ARI = acute respiratory infection, AZ = astraZeneca, BMS = bristol myers squibb, CONNECTS = Collaborating Network of Networks for Evaluating COVID-19 and Therapeutic Strategies, CTSN = Cardiothoracic Surgical Trials Network, CRO = Contract Research Organization, DCRI = Department of Clinical Research Informatics, INSIGHT = International Network for Strategic Initiatives in Global HIV Trials, lilly = eli Lilly and Company, mAbs = monoclonal antibodies, NCATS = National Center for Advancing Translational Sciences, NHLBI = National Heart, Lung, and Blood Institute, NIAID = National Institute of Allergy and Infectious Diseases, OTC = over-the-counter, PETAL = prevention and early treatment of lung injury, PCORnet = National Patient-Centered Clinical Research Network, SGLT2 = sodium-glucose cotransporter-2, TIN = Trial Innovation Network, TRI = Technical Resources International, inc, VA = veterans affairs.

The ACTIV TX-Clin and Preclin WGs evaluated over 800 therapeutic agents with potential application for COVID-19. ACTIV used an intake and assessment process to evaluate and prioritize both preclinical and clinical stage drug candidates for potential inclusion in clinical trials. This process identified 37 candidates (both single and combination drugs, including various dosages and formulations) (Figure 3) that were subsequently evaluated in at least one of the 11 protocol platforms (Figure 4). Detailed timelines for creation, regulatory approval, launch, and operation of these trials shared in Table 1. In aggregate, these protocols enrolled over 26,000 participants.

**Table 1.** Timeline of major master protocol milestones

Master Protocol	Agent	Start Date	First Patient Enrolled Date	Each Interim Analysis by the DSMB Date	Last Patient Enrolled/Trial Closure Date	Date of Last Patient Follow-Up for Primary Endpoint	Date of Pre-Print	Final Date of Publication
**ACTIV-1**	Abatacept	Oct 16, 2020	Oct 18, 2020	Aug 5, 2020 Jan 7, 2021 May 6, 2021 Sept 2, 2021 May 19, 2022	Dec 30, 2021	Feb 26, 2022	Sep 26, 2022	Jul 10, 2023
	Cenicriviroc	Oct 16, 2020	Oct 16, 2020	Aug 5, 2020 Jan 7, 2021 May 6, 2021 Sept 2, 2021 May 19, 2022	Sep 4, 2021	Nov 3, 2021	N/A	Jul 10, 2023
	Infliximab	Oct 16, 2020	Oct 27, 2020	Aug 5, 2020 Jan 7, 2021 May 6, 2021 Sept 2, 2021 May 19, 2022	Dec 17, 2021	Jan 31, 2022	Sep 26, 2022	Jul 10, 2023
**ACTIV-2**	LY-CoV-555	Jul 31, 2020	Aug 19, 2020 (Ph 2) Nov 23, 2020 (Ph 3)	Nov 9, 2020 Apr 26, 2021	Nov 17, 2020 (Ph 2) Jan 15, 2021 (Ph 3)	N/A	N/A	Aug 31, 2023
	BRII-196 + BRII-198	Dec 17, 2020	Jan 5, 2021 (Ph 2) Mar 3, 2021 (Ph 3)	Feb 8, 2021 Apr 5, 2021 Apr 26, 2021 Aug 9, 2021 Aug 23, 2021	Feb 25, 2021 (Ph 2) Jul 30, 2021 (Ph 3)	N/A	N/A	May 17, 2023
	SNG001 (IFN-B1a)	Feb 8, 2021	Feb 10, 2021 (Ph 2)	Mar 22, 2021 Apr 26, 2021 May 24, 2021 Jul 19, 2021 Oct 19, 2021	Aug 18, 2021 (Ph 2)	N/A	N/A	Oct 6, 2023
	Camostat	Jan 29, 2021	Feb 15, 2021 (Ph 2)	Mar 22, 2021 Apr 26, 2021 Jun 14, 2021	Apr 26, 2021 (Ph 2)	N/A	N/A	Oct 5, 2023
	AZD7442 (IM)	Jan 29,2021	Feb 17, 2021 (Ph 2)	Mar 22, 2021 Apr 26, 2021 May 24, 2021	May 20, 2021 (Ph 2)	N/A	N/A	Apr 3, 2023
	AZD7442 (IV)	29-Jan-2021	Feb 24, 2021 (Ph 2)	Mar 22, 2021 Apr 26, 2021 May 24, 2021	May 20, 2021 (Ph 2)	N/A	N/A	Apr 3, 2023
	SAB-185	Apr 9, 2021 (Ph 2) Sep 24, 2021 (Ph 3)	Apr 29, 2021 (Ph 2 low dose) Apr 20, 2021 (Ph 2 high dose) Sep 29, 2021 (Ph 3)	Jul 19, 2021 Sep 20, 2021 Feb 28, 2022	Aug 16, 2021 (Ph 2 low dose) Aug 17, 2021 (Ph 2 high dose) Jan 20, 2022 (Ph 3)	N/A	N/A	Jul 14, 2023
	BMS-986414 + BMS-986413	May 20, 2021	25-May-2021 (Ph 2)	Jul 19, 2021 Sep 20, 2021 Nov 1, 2021	Aug 11, 2021 (Ph 2)	N/A	N/A	N/A
**ACTIV-2D**	Shionogi-217622	Jul 29, 2022	Aug 8, 2022	Dec 13, 2021 Mar 21, 2022 Sep 12, 2022 Jan 30,-2023 May 22, 2023 Sep 14, 2023	Dec 8, 2023	N/A	N/A	N/A
**ACTIV-3**	LY-CoV-555	5-Aug 5, 2020	Aug 5, 2020	Aug 31, 2020 Sep 21, 2020 Oct 26, 2020 Jan 4, 2021	Oct 26, 2020	Feb 1, 2021	N/A	Dec 22, 2020
	Brii-196/Brii-198	Dec 16, 2020	Dec 16, 2020	Jan 4, 2021 Jan 25, 2021 Mar 1, 2021	Mar 1, 2021	Jun 8, 2021	N/A	Dec 23, 2021
	VIR-7831	Dec 16, 2020	Dec 16, 2020	Jan 4, 2021 Jan 25. 2021 Mar 1, 2021	Mar 1, 2021	Jun 9, 2021	N/A	Dec 23, 2021
	AZD7442 (IV)	Jan 29, 2021	Feb 5, 2021	Mar 15, 2021 May 10, 2021 Jun 14, 2021 Jul 19, 2021 Aug 16, 2021 Oct 18, 2021 Nov 15, 2021	Sep 30, 2021	Jan 28, 2022	N/A	Jul 8, 2022
	DARPin MP0240	May 28, 2021	Jun 11, 2021	Jul 19, 2021 Oct 18, 2021 Nov 15, 2021	Nov 15, 2021	Feb 23, 2022	N/A	Aug 9, 2022
	Pfizer PF-07304814	May 28, 2021	Jun 11, 2021	Dec 6, 2021 Jan 10, 2022	Dec 29, 2021	Apr 6, 2022	N/A	N/A
**ACTIV-3B**	Remdesivir	Apr 16, 2021	Apr 21, 2021	N/A	May 25, 2022	Aug 22, 2022	N/A	Jun 19, 2023
	Aviptadil + Remdesivir	Apr 16, 2021	Apr 21, 2021	May 24, 2021 Jun 21, 2021 Aug 16, 2021 Sep 27, 2021 Nov 1, 2021 Dec 13, 2021 Feb 14, 2022 May 25, 2022	May 25, 2022	Aug 22, 2022	N/A	Jun 19, 2023
**ACTIV-4A**	Heparin	Sep 4, 2020	Sep 4, 2020	Dec 16, 2020 Jan 20, 2021	Jan 22, 2021	Jan 25, 2021	N/A	Aug 26, 2021
	P2Y12 Inhibitor	Feb 26, 2-21	Feb 26, 2-21	Jun 16, 2021 Jul 21, 2021 Oct 20, 2021 Dec 15, 2021 Mar 16, 2022 May 20, 2022 Jun 15, 2022	Jun 22, 2022	Jul 6, 2022	N/A	Jan 18, 2022 (moderate cohort) May 1, 2023 (severe cohort)
	Crizanlizumab	Dec 9, 2021	Dec 9, 2021	Aug 17, 2022 Sep 21, 2022	Sep 23, 2022	Oct 12, 2022	N/A	Jun 25, 2023
	SGLT2 Inhibitors	Dec 3, 2021	Dec 3, 2021	Aug 17, 2022 Feb 15, 2023 Mar 27, 2023	Mar 30, 2023	Apr 1, 2023	N/A	N/A
**ACTIV-4B**	Low-Dose Aspirin	Aug 2020	Sep 2020	Jun 18, 2021	Jun 2021	Aug 5, 2021	N/A	Oct 11, 2021
	Prophylactic-Dose Apixaban	Aug 2020	Sep 2020	Jun 18, 2021	Jun 2021	Aug 5, 2021	N/A	Oct 11, 2021
	Therapeutic-dose Apixaban	Aug 2020	Sep 2020	Jun 18, 2021	Jun 2021	Aug 5, 2021	N/A	Oct 11, 2021
**ACTIV-4C**	Apixaban	Feb 9, 2021	Feb 15, 2021	Oct 20, 2021 Apr 20, 2022 Jun 15, 2022	Jun 23, 2022	Sep 24, 2022	N/A	Mar 21, 2023
**ACTIV-4HT**	TXA127 (Constant)	Jul 14, 2021	Jun 22, 2021	Apr 20, 2022	Apr 14, 2022 Apr 20, 2022	May 13, 2022	N/A	Apr 11, 2023
	TRV027 (Trevena)	Jul 14, 2021	Jun 27, 2021	Apr 20, 2022	Apr 15, 2022 Apr 20, 2022	May 13, 2022	N/A	Apr 11, 2023
	Fostamatinib	Nov 11, 2021	Nov 17, 2021	Jan 18, 2023	Sep 27, 2023 Sep 27, 2023	Oct 25, 2023	N/A	N/A
**ACTIV-5**	Risankizumab	Oct 14, 2020	Jul 13, 2021	May 7, 2021	Jul 13, 2021	Sep 15, 2021	N/A	N/A
	Lenzilumab	Oct 19, 2020	Oct 23, 2020	Aug 9, 2021	Jan 9, 2022	Apr 22, 2022	N/A	Jul 6, 2022
	Danicopan	Jul 21, 2021	Feb 21, 2022	Aug 9, 2021	Feb 21, 2022	Apr 23, 2022	N/A	N/A
**ACTIV-6**	Ivermectin 400	Jun 11, 2021	Jun 23, 2021	Nov 18, 2021	Feb 4, 2022	Mar 15, 2022	Jun 12, 2022	Oct 21, 2022
	Fluticasone	Aug 6, 2021	Aug 9, 2021	Feb 24, 2022	Feb 9, 2022	Mar 17, 2022	Aug 11, 2022	Sep 21, 2023
	Fluvoxamine 50	Aug 6, 2021	Aug 6, 2021	Feb 24, 2022	May 27, 2022	Jul 4, 2022	Nov 1, 2022	Jan 12, 2023
	Ivermectin 600	Feb 16, 2022	Feb 17, 2022	June 22, 2022	Jul 22, 2022	Aug 29, 2022	Dec 16, 2022	Feb 20, 2023
	Fluvoxamine 100	Aug 25, 2022	Aug 30, 2022	Dec 19, 2022	1/20/23	Feb 27, 2023	Sep 13, 2023	Nov 17, 2023
	Montelukast	Jan 27, 2023	Jan 27, 2023	Apr 25, 2023	Jun 23, 2023	Aug 3, 2023	N/A	N/A
	Metformin	Sep 5, 2023	Sep 18, 2023	N/A	N/A	N/A	N/A	N/A

ACTIV = Accelerating COVID-19 Therapeutic Interventions and Vaccines.

ACTIV opines that the selection and prioritization process could be improved by the following modifications: 1) pre-developing target product profiles (TPPs) for agents for pandemic potential viruses; 2) better integrating big data resources to incorporate real-world data, e.g., especially when considering repurposing agents with mechanism of action similar to proven effective agents; incorporating high-throughput screening techniques and data from them to have additional candidate therapeutics to be evaluated as the disease pathogenesis is better understood; 4) tracking viral evolution and emergence of new viral variants [6]; and 5) aiming to incorporate evaluation of therapeutics effects across post-acute sequelae symptoms that could arise, such as those for SARS-CoV-2 [7]. The prioritization process could also benefit from more integrated data sharing and product prioritization from all efforts, such as those from other global platform trial efforts, Coronavirus Immunotherapy Consortium [8] and the Bill and Melinda Gates Foundation during the COVID-19 pandemic.

## Preclinical testing

The pandemic required private and public research organizations to pause their primary efforts (and in some cases their missions), address the immediate threat, and focus on identifying the most promising anti-SARS-CoV-2 drug candidates to be tested in clinical trials. During preclinical development, organizations endeavored to collect and share sequences for the novel viral variants; employed high-throughput screening mechanisms to identify potential direct acting anti-viral candidates from portfolios of approved medications and diverse chemical libraries; isolated candidate neutralizing monoclonal antibodies (mAbs) from survivors of COVID-19; or proposed anti-inflammatory compounds to control the sepsis-like disease pathogenesis. Committees were established to prioritize the compounds to go into animal testing and transition to clinical development.

While animal models were preferred for preclinical efficacy testing, they required available level 3 and 4 biosafety laboratories (BSL) be identified and adequate animal colonies be established to test top candidates. TPPs were created for mAbs and direct acting small molecules but had to be refined as disease information increased. As testing progressed, misaligned priorities in the PPP required more animals be used per study and further delayed *in vivo* testing of therapeutic candidates. Below these challenges are explored and general recommendations made.

### Harmonization and rapid dissemination of viral sequences and live virus reference reagents

The first therapeutic and vaccine candidates were identified within days of access to the SARS-CoV-2 viral sequence of Wuhan strain in early 2020, illustrating the importance having immediate access to the viral sequence. However, as the virus adapted to its human host, access to the sequences of new variants was delayed due to several factors: global capacity insufficient to meet the urgent need to sequence thousands of clinical isolates; absence of common and consistent workflows for generating consensus sequences and for sharing those sequences; and regulatory requirements that delayed sharing of live viral isolates, all substantially slowing preclinical testing as individual groups had to culture or replicate the virus. Continued laboratory assessment of the efficacy of agents against newer strains was often delayed until the new strain was detected in clinical specimens collected in the USA. The ACTIV PPP worked together to build capacity and to harmonize the processes for generating consensus viral sequences and sharing (e.g., Global Initiative on Sharing Avian Influenza Data [GISAID] and International Nucleotide Sequence Database Consortium [INSDC]).

The pandemic response required rapid testing, which in turn relied on the rapid dissemination of reference live virus strains (to verify test performance) and on dependable reagents and materials (to perform the tests). Dissemination of viral isolates to laboratories across international borders was often far slower (>one month) than the clinical spread of each new variant. The delay hampered the research community’s ability to keep up with generation of new approaches to emerging variants. The National Institutes of Health (NIH) supported an effort, through Biodefense and Emerging Infections (BEI) and the American Type Culture Collection (ATCC), to provide sequence-verified viral stocks for research and development. The response to future pandemics will demand rapid assessment (and likely augmentation) of the international capacity to sequence clinical isolates, harmonize mechanisms to generate consensus sequences, and post them to sites available to researchers. Assays for the effectiveness of proposed agents using live virus in cell culture and in animal models are essential to prioritization of those agents for further laboratory study and clinical trials. Results of these studies weighed heavily in every prioritization effort, ACTIV and others. Lack of a harmonized protocol for live virus testing was a barrier to antiviral development. ACTIV provided written guidance on *in vitro* and *in vivo* standards assays to help point to important steps in *in vitro* and *in vivo* testing systems [9,10]. International agencies and governments should develop an emergency protocol for accelerating the exchange of crucial materials. Finally, as much as is possible, viral stocks should be distributed equitably to laboratories across the globe to ensure fair and reasonable access to testing of agents. This reasonable access will be essential to ensure diversity of thought and enhance innovation and rapid response.

### Biosafety level testing facilities

Due to SARS-CoV-2’s infectivity and lethality, *in vitro* and *in vivo* testing required laboratory facilities designed and operated to enhance biosafety. Many investigators did not have access to these facilities and did not know how to locate them. The ACTIV Preclin WG collected lists of BSL-3 and -4 to conduct *in vitro* assays and animal studies [11], and found that access to enhanced biosafety facilities depended on whether the requesting organization was private versus public or required an academic collaboration. The ACTIV team helped match research needs with facility capabilities, but often found that the volume of work exceeded the capacity of the existing facilities. Capacity, as well as project prioritization for BSL3/4 testing, should be addressed for future emergencies.

### Antiviral testing assays and models

Animal testing was difficult and delayed for multiple reasons: it was difficult to establish a consistent and reliable animal model corresponding to the human disease; inconsistent models for severe disease; lack of reference standards for cross comparison analysis; nonharmonized challenge and treatment protocols; and the lack of clinical validation. Coordination of access to animals to ensure the most promising compounds were tested first would have been useful. Better understanding of which animal models may be appropriate for testing pandemic potential viruses anticipated to cause future pandemics would be useful, as would maintenance of breeding colonies of these animals to permit immediate testing after virus identification. In addition, BSL-3 laboratories should be enlarged to permit testing larger numbers of animals and to house necessary histopathology equipment. Finally, animal testing protocols require harmonization, e.g., dose and administration route, to eliminate delays during protocol re-versioning and to reduce the number of animals tested per therapeutic candidate.

### Expediting communication of preclinical results to the prioritization committees

Rapid data sharing was an ACTIV priority and was addressed by the ACTIV Open Data Portal (ODP). As more groups began working on COVID-19, new results reports numbers grew faster than most research and clinical investigators and organizations could assimilate. This information overload provided the impetus for parallel data compilation websites like the ACTIV ODP. Each website focused on areas of interest to the founding group. For ACTIV, the Tracking Resistance And Coronavirus Evolution (TRACE) group was asked to focus on therapeutic efficacy in *in vitro* assays for therapeutics of interest and vaccine sera to emerging variants [12]. Initially, ACTIV ODP data came from published scientific journal results that used confirmed viral sequence variants. Eventually, ACTIV’s industry partners sent internally curated data prior to publication. In the end, a large percentage of *in vitro* efficacy testing for COVID-19 therapeutics data was submitted prior to publication.

Resources related to therapeutic testing and ODP results can be found on the ACTIV ODP webpage. This website was adopted as a place where ACTIV, TRACE, and any NIH resource link could be placed to make information easier to find by researchers, while supplying data analysis capabilities. The data have been maintained in downloadable format, so detailed analysis can be conducted outside the site. In this way, the website serves the widest communities of researchers interested in tackling pandemic issues. In the future, websites such as ACTIV ODP will be critical to provide links to similar tools with different focus areas, allowing researchers to focus on data analysis and not finding data.

In addition to websites like the ACTIV ODP, the next pandemic response effort modeled on ACTIV should consider hosting a dedicated preprint server (or using a reputable preprint server) and implementing a supporting data server for rapid data sharing that would allow for early and interim results to be posted for researchers participating in the PPP, as well as the rest of the scientific community after the DSMB review.

## Master protocol design

The majority of recommendations for early master protocol design decisions can be found in the ACTIV TX-Clin WG’s first manuscript [4]; additional lessons learned for protocol design as the pandemic progressed are captured here.

### Establish efficient master protocols

In retrospect, evaluation of investigational therapeutics by ACTIV could have been accomplished with fewer than the 11 master protocols (Figure 4 and Table 1). Throughout the pandemic, ACTIV evolved so there are now only three master protocols: a trial for inpatients (STRIVE), a trial for outpatient (ACTIV-2), and an outpatient fully remote trial (ACTIV-6). For future pandemics, three master protocols similarly modeled will allow enough patient population flexibility, trial design rigor, and desired amount of clinic-based care to allow for evaluation of a full spectrum of therapeutic candidates. These protocols should be written to allow for expansion cohorts of more vulnerable populations, such as long-term facility residents, children, and pregnant women, once the regulatory agencies feel the data are sufficient to demonstrate safety to warrant their inclusion. Inclusion of these populations in the ACTIV trials was a major challenge for the partnership. This should be changed in future emergency efforts.

For outpatient trials, ACTIV recommends a single protocol be used for testing of any novel or repurposed agents for which there is an intent to file for registrational approval. This protocol could also allow for flexibility for more pragmatic approaches to expedite testing when regulators and companies with agents being tested agree. In addition, to make the master protocol more efficient for rapid drug assessment, it should be constructed as a Phase 2/3 progressive design, as proved effective for ACTIV-2.

A third master protocol found to be of critical importance for ACTIV was a fully remote, highly pragmatic, decentralized trial for testing repurposed agents in diverse outpatient populations, which manifested as ACTIV-6, the last master protocol launched. This protocol design proved to be best suited for evaluation of repurposed agents with extensive safety profiles and simple administration routes allowing for delivery of investigational products directly to participants’ homes for self-administration. This design permits rapid initiation and testing of widely available agents of high interest to both the scientific and general community. Although the intent is to have a simple design with limited data collection and no specimen collection, the protocol should allow for some rigor such as blinding and placebo controls for evidence generation for informing clinical guidelines. Ideally, this should be one of the first trials to be initiated at the start of any emergency to drive rapid evaluation of repurposed agents.

### Engage regulators early

Based on the ACTIV experience, future master protocols teams should work for alignment across regulators during peacetime or within the first weeks of a public health emergency (PHE), including FDA, the European Medicines Agency (EMA), and others across the globe, on a set of clear clinical endpoints that can evolve with the changing nature of the disease or emergency. While many inpatient trials initially used the WHO Ordinal Scale for endpoints, as the COVID-19 pandemic evolved, some trials needed to modify this scale to better classify patients, and outpatient trials eventually found endpoints used early in the pandemic (e.g., hospitalization and death) became insufficient for subsequent trial conduct due to decreasing severity of disease and increased efficacy of symptom-mitigating care. It became necessary to work with the regulatory agencies to agree upon progressive, innovative composite endpoints that included symptomatic assessments and patient reported outcomes. For future protocols, it would be beneficial to have a set of pre-approved endpoints accounting for disease evolution, but reliable enough for protocol use in non-emergency times.

## Master protocol implementation

Designing master protocols to address public health emergencies involves grappling with complicated scenarios and making numerous decisions regarding clinical trial elements. Protocol design and protocol implementation have distinct hurdles and challenges; the former must be overcome before the latter can be addressed. Each ACTIV trial had its own operational hurdles, but many shared commonalities which are enumerated here.

### Improve international regulatory coordination

Receiving regulatory approvals is a rate-limiting step to clinical trial initiation, and particularly so in global trials where every national regulatory authority reviews. The ACTIV protocol teams’ experiences with different regulatory bodies were mixed. For the first ACTIV protocols to be launched, teams worked closely with the FDA on trial design issues and frequently communicated with FDA staff during trial implementation. FDA review periods varied by division, in some divisions the ACTIV teams communicated regularly with FDA often solving issues before reviews which were often well under 30 days at the start of the PHE, leading to short development periods before trial launch at US sites. (ACTIV timelines for FDA review in Supplemental Figure 1.) Other FDA divisions were less open to communication, and approvals were slower. Review by global non-US national regulatory bodies suffered from sequential submissions was quite protracted and in some cases international sites could not meaningfully contribute to the trials. A global regulatory committee should be established to facilitate simplification and harmonization prior to the next pandemic to facilitate the rapid development and implementation of trials when the next public health emergency is declared. The World Health Organization has taken a step in this direction by establishing an international regulatory working group as part of the recent First Global Clinical Trials Forum, November 20-21, 2023.

### Establish a United States Government (USG) prioritized clinical research agenda

ACTIV was initiated in April 2020 and launched its first master protocols in July, fast by non-emergency timelines. Despite this rapid start, ACTIV did not start simultaneously with the pandemic declaration. It took from April to August 2020 to implement the first master protocols, and in the meantime many clinical trial sites were already implementing a myriad of other COVID-19 therapeutic trials. (ACTIV sub-study development and launch timelines in Table 1 and Supplemental Figure 1.) Most of these trials were not large enough to provide actionable data [15], albeit with a few notable exceptions, such as Adaptive COVID-19 Treatment Trial (ACTT). The numerous small trials ultimately led to delays in master protocol implementation as site staff were stretched thin with clinical care responsibilities and multiple clinical trials. In future pandemics, it is critical that clarity exists on which USG entity is the lead for research response, establishing upfront the clinical research agenda, including for known pathogens in “peace time,” and that entity, in turn, must make clear which trials should be prioritized. Financial incentives and other mechanisms, such as required terms in grants/contracts to clinical sites, should also be considered to encourage or require sites to participate in higher priority trials.

### Maintain clinical trial infrastructure for future pandemic response

To further aid in rapid trial implementation, global, activation-ready clinical trial networks should be identified and maintained for future pandemics. Engagement of established clinical trial networks performing other clinical research, such as those for HIV or respiratory illness treatment, efficiently jumpstarted the ACTIV master protocols. Networks can be kept “warm” by continuing to study COVID-19 treatments and/or by studying related conditions (e.g., influenza or other respiratory infections).

Based on this vision, ACTIV established in 2022, a single global platform trial entitled, Strategies and Treatments for Respiratory and Viral Emergencies (STRIVE) [13,14]. STRIVE was launched by a network formed by combining all the inpatient ACTIV networks developed from combining and streamlining the ACTIV-1, −3/3B, and −5 trial networks. STRIVE has been implemented by 300+ sites, 61% of which are in LMICs, on 6 continents. The platform is structured so trials with registrational intent, as well as more pragmatic/strategy trials, can be conducted depending on population needs and agent profile. The first two trials in STRIVE are focused on COVID-19 and funded by residual funds from Operation Warp Speed, but the platform is intended to address any pandemic-causing pathogen and will seek new types of funding mechanisms as new trials are initiated. In fact, STRIVE has pivoted to not only being a standing protocol but also a full network with capabilities to take on trials beyond the initial protocol, including outpatient studies. By having a standing, active protocol and trial infrastructure, such as STRIVE, new trial startup time for novel pathogen treatment evaluation will be greatly decreased from 2 to 3 months to just weeks.

Since beyond hospital use of remdesivir, the current licensed vaccines and direct antivirals were FDA approved on basis of outpatient trials, networks active with other primary outpatient research should be encourage to remain “on call” and build accelerated pivoting capacity, in preparation for the next pandemic. Additional sites should be identified, in advance of a PHE, to engage populations not reached by standing research sites, e.g., different geographic regions serving populations with varied race/ethnicities, socioeconomic class, age, and place of residence (e.g., nursing homes) to promote diversity, equity, inclusion, and access to trials to all participants.

### Promote inclusion of diverse patient populations

The ACTIV trials faced challenges in recruiting diverse populations. Importance of clinical trial site settings, global recruitment, targeted messaging to specific populations and geographic areas alongside general information via the combatcovid.com website, and misinformation counteraction ACTIV attempted to recruit and engage diverse and underserved populations in accordance with good participatory practices [16]. While the ACTIV trials included community advisory boards, community inclusion in preparedness activities will enable greater awareness, interest alignment, and emergency research support. Engagement of a broad cross-section of people globally through outreach and participation in the design and planning of the response to the next global health threat will be essential to bolster trust and uptake of research results [16]. Stronger engagement will create sufficiently large sample subpopulations for data analyses resulting in reliable information for a broader population. This will be strengthened by implementing a minimal number of platform trials, testing new interventions in a large number of participants with adequate representation of geographical and population setting, disease severity, and participants with diverse characteristics defined by race/ethnicity, sex, gender identity, and age as recommended by the current FDA guidance on diversity plans for clinical trial enrollment [17].

## Results communication and data sharing

### Develop consensus data format

A critical goal for therapeutic clinical trials is to rapidly deliver high-quality data to meet regulatory standards for new drug applications. Several data collection challenges, management practices, and deliverables arose across the ACTIV trials. Without a common electronic health record (EHR) reporting system, hospitals resorted to faxing occupancy data during early days of the COVID-19 pandemic, and researchers relied on the Johns Hopkins University COVID-19 tracker [18], a rapidly developed, useful tool whose information nonetheless inevitably lagged about changing disease dynamics due to overwhelmed clinical sites having to report results in nonefficient ways. This state of affairs undermined trial planning and calls for regulation requiring timely and accurate data reporting into a common system during PHEs. This delayed information led to a lack of standardized primary trial endpoints for different populations and delay in evolution of clinical outcomes over time decreasing uniform data collection. Logistic challenges included a need for a mechanism for centrally located CRO personnel to reach into the EHR and other records to assess recruitment, audit adherence, collect outcomes and provide guidance to onsite personnel, site staffing limitations, data queries could only be resolved asynchronously, and responses were often delayed, and data cleaning and monitoring variation across ACTIV trials.

### Mandate central data and sample repositories

Data sharing efforts were hindered in ACTIV trials due to cumbersome data management systems imposed by existing procedures at contract research organizations (CROs) and a lack of coordination of data management approaches across trials. Language about data sharing should be incorporated into protocols and informed consent forms. Data structure formats and transfer timeline should be agreed upon by all stakeholders during trial design. Coming to agreement over these issues and developing data collection formats during pre-pandemic planning will ensure rapid implementation during the next PHE, while designing systems flexible enough to adapt to changes during a pandemic.

Other useful data tools essential to a speedy PHE response would be a standard Statistical Analysis Plan that could be lightly customized to the particulars of the pathogen and to the illnesses/threats and predefined and constructed data capture infrastructure complying with current NIH data sharing agreements but accelerate sharing.

A central repository for biospecimens connected to the phenotyping data from all master protocols should also be established, creating an ability for researchers to quickly access and analyze samples for validation of newly emerging scientific hypotheses or quick validation of potential biomarkers. Extensive experience from ACTIV trials provides a framework for developing consensus data and biospecimen management practices and collection for this central repository for future pandemic trials.

### Develop a strong communication and results dissemination plan

To help address rapid disease evolution and need for clinical trials to continuously adapt, each master protocol should be designed and resourced so data from each trial arm can be prepared and released within four weeks (or sooner) from final patient primary endpoint follow-up visit. This will allow positive and negative agent data to be shared with regulators and guidelines committees to change clinical practice rapidly. In addition, it will help other trials track changing demographics and provide them an ability to pivot their trial design, power calculations, or endpoints to keep trial results relevant. These resources allowing for rapid results dissemination should be one component of an overarching communications plan that is prepared and implemented at the start of each master protocol.

### Manufacturing and drug scaling

ACTIV’s charge was to identify and test therapeutic strategies to be deployed, at scale, with historic rapidity without sacrificing safety or rigor. Considerations around practicalities of implementation entered the earliest stages of discussion of candidate therapeutics. Some assessment of supply, manufacturing capacity, and logistical barriers was built into the ACTIV agent prioritization process [3].

### Allocate funds for drug supply and manufacturing capacity

Many compounds submitted for evaluation did not have adequate drug supply for animal testing or for advancement into human clinical trials. During COVID, funding streams were imperfectly aligned for manufacturing to support research, IND-enabling, and clinical studies. Public funds were routed through the Biomedical Advanced Research and Development Authority, which was also tasked with managing other critical portfolios such as vaccine development. US contract manufacturing facilities rapidly reached their maximum capacity. The requirement for US manufactured products resulted in delayed testing even for companies with available drug if it was manufactured ex-US. For future pandemics, funds for manufacturing study drugs should be included and greater flexibility in (global) sites of manufacture could help reduce supply delays.

### Align selection criteria for supply with therapeutic priorities

Given the overload on manufacturing capabilities, considering large-scale availability of candidate therapies early in agent prioritization creates tension. A promising therapy for a novel potential pandemic pathogen may be at a stage of development that has not yet reached a need for scalability (e.g., biologics vs. small molecules or experimental vs. marketed). Future pandemic response efforts could be accelerated by closely mapping manufacturing resources to maintain the viability of early therapeutic options without implementation concerns.

### Designate resources and strategies

Manufacturing, scaling, and distribution of therapeutics involve an intricate and interconnected network subject to factors that include (but are not limited to) availability of functional production facilities, complicated contractual relationships, negotiated license agreements, supply and transport chain stability, and other legal arrangements typically optimized for non-emergency conditions. To be prepared in these scenarios, the US Administration for Strategic Preparedness and Response maintains the Strategic National Stockpile (SNS) [19] as a medical response stopgap resource. Future responses to pandemics may be accelerated if procedures could be established in advance for accessing therapies available to be included in USG-supported trials.

### Minimally restricted agent access

Another specific opportunity identified for maximizing the power of the PPP structure for future pandemic responses is to prepare agreements, contracts, and procedures before the next pandemic, particularly with private sector partners that allow for continued clinical research on EUAs enabled USG SNS agents. Building the “how” and “when” infrastructure beforehand with a future PPP in mind would minimize manufacturing, scaling, and distribution delays.

## Conclusion

All lessons captured in these therapeutic development areas were derived from what the ACTIV PPP accomplished for the COVID-19 response and are summarized in the recommendations in Figure 2.

In addition, a final lesson was taken from the work of ACTIV. Trust is the most important element for any public health response, whether a pandemic or usual care, and a PPP can be a method of building such trust. One advantage of forming a PPP is each participant has a voice in shaping the solution to a challenge. Each partner brings unique strengths to the PPP and the partnership can be designed to capitalize on them, such as government’s capability to leverage public infrastructure and funding, industry’s capacity to manufacture, academic investigators’ ability to conduct rigorous clinical studies, and community-based organizations established and long-standing relationships with targeted groups. Playing to these strengths allows each partner to shine and engenders trust across the PPP and broader health community. The PPP structure and intent to collaborate also allows for ongoing and free exchange of information which, during the COVID-19 response, benefited all stakeholders.

The ACTIV PPP showed many stakeholders could come together quickly and efficiently, assembling the initial partnership governance in less than a week, to participate in unselfish dialogue and advance solutions in a major health crisis. The model and lessons learned during this PHE should be translated into best practices for future pandemics, as well as in the conduct of ordinary healthcare and to revolutionize science. While the authors realize that large collaborations to aid therapeutic development during non-PHE times can be challenging, such as the ones facilitated by the FNIH has facilitated examples of these peacetime drug development collaborations, such as the Accelerating Medicines Partnerships [20,21], Lung-MAP [22–24], and projects within the Biomarkers Consortium [25,26].

One component of this multistakeholder engagement that will need to be conducted both in peacetime and during the next PHE is community stakeholder engagement. ACTIV did this through multiple outlets, including the NIH Community Engagement Alliance and Combat COVID [27]; however, both efforts were started during the pandemic, and although both proved successful, they did not have the full reach desired into underserved communities. This type of engagement is discussed more fully in the Outreach, Recruitment, and Engagement manuscript in this special issue. Overall, ACTIV hopes this manuscript provides an executive perspective of key lessons and recommendations for future PHE responses. While many deeper tactical lessons were learned from the ACTIV trials exist, this information will be captured in subsequent reports assembled by the ACTIV master protocol teams.

## Supporting information

Adam et al. supplementary material Adam et al. supplementary material

## Supplementary material

The supplementary material for this article can be found at https://doi.org/10.1017/cts.2024.178.
