# Supplementary figures and images for: Preparing better: Accelerating COVID-19 Therapeutic Interventions and Vaccines (ACTIV) therapeutics trials lessons learned: A call to the future
Source: J Clin Transl Sci. 2024 Oct 15;8(1):e150. doi: 10.1017/cts.2024.178 (PMC11523022; doi:10.1017/cts.2024.178)

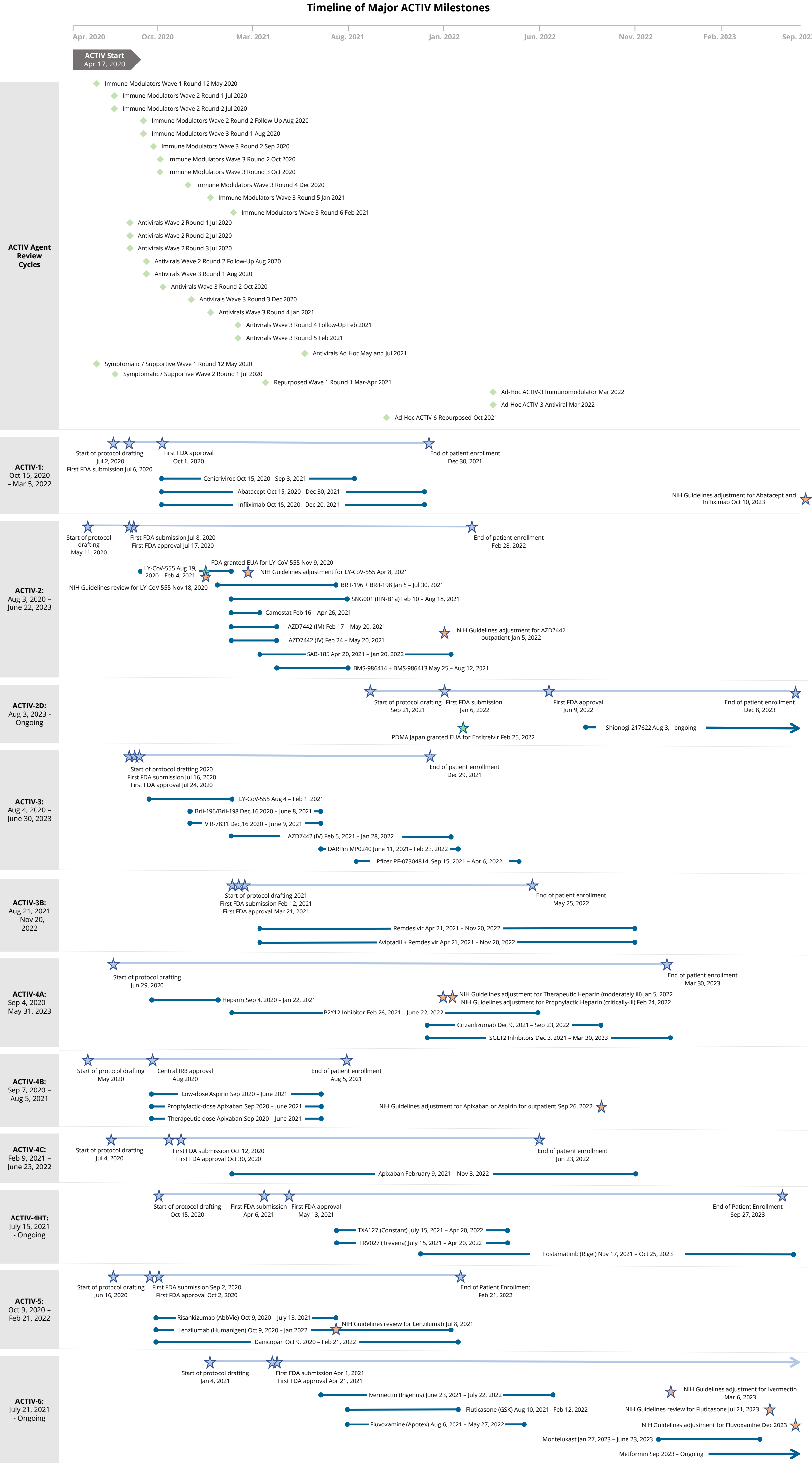

Supplement: Adam et al. supplementary material [file S205986612400178Xsup001.pdf]
